# Synergistic Enhancement of Rhodamine B Adsorption by Coffee Shell Biochar Through High-Temperature Pyrolysis and Water Washing

**DOI:** 10.3390/molecules30132769

**Published:** 2025-06-27

**Authors:** Xurundong Kan, Yao Suo, Bingfei Shi, Yan Zheng, Zaiqiong Liu, Wenhui Ma, Xianghong Li, Jianqiang Zhang

**Affiliations:** 1 College of Materials and Chemical Engineering, Southwest Forestry University, Kunming 650224, China; kanxurundong@163.com; 2 International Union Laboratory of China and Malaysia for Quality Monitoring and Evaluation of Agricultural Products in Yunnan, School of Biology and Chemistry, Pu’er University, Pu’er 665000, China; 15125343559@163.com (Y.S.); shibingfeipexy@163.com (B.S.); zhengyan@peu.edu.cn (Y.Z.); lzq15025110725@126.com (Z.L.); 3 Modern Engineering College, Yunnan University, Kunming 650000, China; mawenhui@ynu.edu.cn

**Keywords:** coffee shell biochar, water washing, Rhodamine B adsorption, regeneration capacity, sustainable wastewater treatment

## Abstract

Biochar-based adsorbents synthesized from agricultural wastes have emerged as economical and environmentally sustainable materials for water purification. In this study, coffee shell-derived biochars were synthesized via pyrolysis at 500 and 700 °C, with and without water washing, and comprehensively characterized to evaluate their potential for removing Rhodamine B (RhB) from aqueous solution. Structural and surface analyses indicated that a higher pyrolysis temperature enhanced pore development and aromaticity, whereas water washing effectively removed inorganic ash, thereby exposing additional active sites. Among all samples, water-washed biochar pyrolyzed at 700 °C (WCB700) exhibited the highest surface area (273.6 m^2^/g) and adsorption capacity (193.5 mg/g). The adsorption kinetics conformed to a pseudo-second-order model, indicating chemisorption, and the equilibrium data fit the Langmuir model, suggesting monolayer coverage. Mechanism analysis highlighted the roles of π–π stacking, hydrogen bonding, electrostatic interaction, and pore filling. Additionally, WCB700 retained more than 85% of its original capacity after five regeneration cycles, demonstrating excellent stability and reusability. This study presents an economical approach to valorizing coffee waste as well as provides mechanistic insights into optimizing biochar surface chemistry for enhanced dye removal. These findings support the application of engineered biochar in scalable and sustainable wastewater treatment technologies.

## 1. Introduction

The rapid development of industrial and agricultural activities has recently resulted in a considerable increase in the discharge of organic pollutants into aquatic ecosystems, presenting serious environmental and health risks [1]. Rhodamine B (RhB), a cationic xanthene dye that is widely used in the textile, paper, and food industries, has attracted attention because of its toxicity, mutagenicity, and resistance to biodegradation [2,3]. Therefore, effective and sustainable strategies for the removal of RhB from water bodies are urgently required.

Among the available treatment methods, adsorption is notable owing to its operational simplicity, high efficiency, and cost-effectiveness [4,5]. Biochar, a carbon-rich porous material produced through the pyrolysis of biomass under limited oxygen conditions, is an effective, sustainable adsorbent owing to its large surface area, functional surface groups, and chemical stability [6,7]. Numerous studies have demonstrated the potential of biochar for dye removal, including RhB, through mechanisms involving pore filling, π–π interactions, hydrogen bonding, and electrostatic attractions [8,9,10].

Valorizing coffee waste is of increasing importance from the perspective of the circular economy, especially considering the widespread global consumption of coffee. Coffee waste, such as coffee shells, is typically discarded, yet it holds significant potential as a raw material for various applications, including biochar production. By converting this agricultural by-product into biochar, we not only address waste disposal but also create a valuable material that can be used in environmental applications like dye removal. This process aligns with the growing demand for sustainable solutions in both waste management and environmental remediation.

Agricultural byproducts such as coffee husks are increasingly being explored as renewable feedstocks for biochar production. Coffee is one of the most widely consumed beverages globally and generates substantial quantities of solid waste in the form of husks, peels, and shells [11]. The conversion of these residues into functional biochars provides an environmentally sustainable method of waste valorization while generating value-added materials for water purification [12,13]. Coffee shell-derived biochar (CSB) exhibits excellent adsorption properties for organic dyes, including RhB, owing to its unique pore structure and aromatic surface functionalities [14,15]. Although some studies have discussed the effect of biochar on dye removal, few studies have systematically explored the synergistic effects of pyrolysis temperature and water-washing treatment on the adsorption performance of biochar. This study aims to fill this research gap by optimizing the pyrolysis temperature and exploring the influence of water-washing treatment on the adsorption performance of biochar.

The primary goal of this study is to valorize coffee shell waste by converting it into biochar through pyrolysis and post-treatment washing. This approach not only addresses the issue of coffee waste disposal but also provides a valuable material for environmental applications such as dye removal, enhancing the sustainability and cost-effectiveness of wastewater treatment processes. The novelty of this study lies in optimizing pyrolysis temperature and washing conditions to improve the biochar’s adsorption properties.

Coffee shell-derived biochar (CSB) primarily consists of lignocellulosic materials such as cellulose, hemicellulose, and lignin, with small amounts of ash and other minerals. CSB is obtained from the hard outer shells of coffee beans, which are typically discarded as agricultural waste during coffee production.

Despite these promising results, several knowledge gaps remain. First, the effects of the pyrolysis conditions, particularly temperature and residence time, on the physicochemical structure and adsorption behavior of CSB have not been systematically investigated [16]. Second, the adsorption mechanisms of RhB onto CSB, including the role of specific surface functional groups and mineral content, remain poorly understood [17]. Third, although the performance of CSB in ambient aqueous systems has been reported, its behavior under extreme environmental conditions, such as high salinity, low temperature, and acidic or basic pH, requires further exploration [18].

Additionally, the development of multifunctional or smart biochar-based composites such as magnetic, stimuli-responsive, and polymer-modified materials provides innovative methods for improving the selectivity, adsorption rate, and recyclability of biochar systems [19]. Such advanced materials may enable real-time monitoring or self-regulation of adsorption performance, which aligns with the emerging trends in precision environmental remediation [20].

We hypothesized that by combining higher pyrolysis temperatures with water washing, pore accessibility could be maximized and RhB adsorption promoted by increasing the specific surface area and removing ash components (which hinder adsorption).

Therefore, this study aimed to (1) synthesize and characterize coffee shell-derived biochars under various pyrolysis and post-treatment conditions; (2) evaluate their adsorption efficiency toward RhB; (3) analyse the adsorption kinetics, isotherms, and mechanisms; and (4) assess the reusability and stability of the optimized biochars. This study contributes to the rational development of cost-effective, scalable adsorbents for sustainable water treatment.

## 2. Results and Discussion

### 2.1. Material Characterization

To elucidate the physical and chemical properties of coffee-shell-derived biochar, a combination of morphological, textural, structural, and chemical analyses was conducted using scanning electron microscopy (SEM), Brunauer–Emmett–Teller (BET), X-ray diffraction (XRD), and Fourier transform infrared spectroscopy (FTIR) techniques. These characterizations aimed to clarify the influence of the pyrolysis temperature and post-washing treatment on the structure and adsorption behavior of the biochar.

#### 2.1.1. SEM Analysis

**Figure 1:**
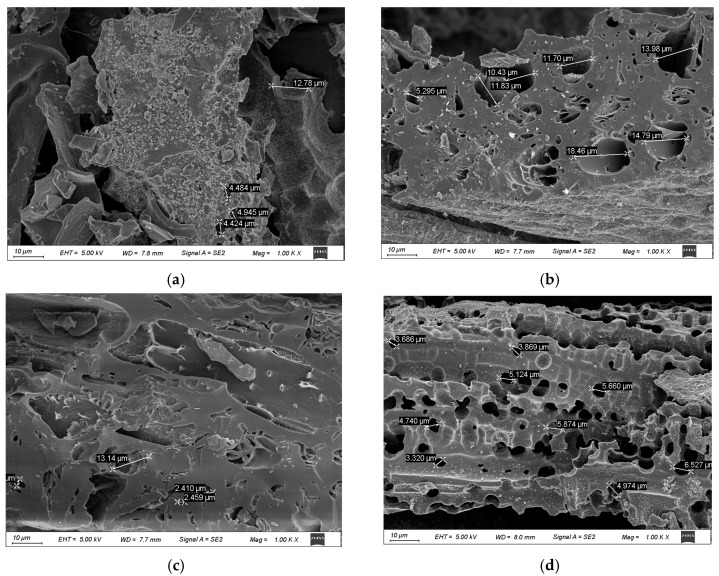
Scanning electron microscopy images of coffee shell biochar at different pyrolysis temperatures and washing conditions. Scanning electron microscope images show the surface morphology of coffee shell biochar under different pyrolysis temperatures (500 °C, 700 °C) and washing treatments (WCB500, WCB700). (**a**,**b**) display CB500 and CB700, respectively, while (**c**,**d**) show WCB500 and WCB700. It can be observed that there are significant changes in pore structure and surface roughness due to differences in pyrolysis temperature and post-treatment washing.

The surface morphologies of the biochar samples prepared at different pyrolysis temperatures with and without water washing were examined using SEM, as shown in Figure 1a–d. Pronounced variations in the surface texture and pore development were observed, highlighting the influence of thermal treatment and post-processing on the structural characteristics of the biochar.

CB500 (Figure 1a) exhibited a relatively dense and compact morphology with minimal porosity. The surface remained largely intact and retained characteristics similar to the original coffee shell biomass, indicating limited structural decomposition at 500 °C. The limited formation of pores is attributed to insufficient thermal degradation of cellulose, hemicellulose, and lignin components at this temperature, which suppresses the evolution of volatile matter and restricts pore generation [21].

In contrast, CB700 (Figure 1b) showed a notably more disrupted and fragmented surface, characterized by a visible increase in pore density and surface roughness. The enhanced morphological evolution was attributed to more extensive devolatilization and aromatization during high-temperature pyrolysis, which facilitates the formation of a porous carbonaceous framework [22]. However, the presence of surface deposits, possibly composed of inorganic ash residues, indicates the partial blockage of active sites and internal porosity [23].

After washing with water, WCB500 (Figure 1c) exhibited moderate morphological improvement. The surface exhibited a rough texture, with localized pores becoming discernible, indicating the effective removal of loosely bound ash and soluble inorganic compounds [24]. However, the limited porosity remained owing to the inherently minimal structural breakdown at 500 °C.

WCB700 (Figure 1d) exhibited a highly porous, sponge-like morphology, characterized by interconnected macropores and an expanded surface area. The elimination of surface-bound mineral phases during washing further exposed the pore network and enhanced the accessibility of the internal adsorption sites [25]. This sample exhibited the most favorable structural features, indicating a synergistic enhancement of pore development through high-temperature pyrolysis and post-treatment, which is critical for optimizing the adsorption performance in aqueous environments [26].

#### 2.1.2. BET Surface Area and Pore Structure Analysis

To further elucidate the pore structure of the biochar samples, nitrogen adsorption–desorption isotherms were measured, and the BET method was employed to determine the specific surface area, total pore volume, and average pore size following standardized protocols for gas adsorption analysis [27]. Table 1 presents the primary influence of pyrolysis temperature and post-treatment on the textural properties of biochar.

The pyrolysis temperature notably influenced the surface area and porosity. At 500 °C (CB500), the biochar exhibited a modest specific surface area of 56.3 m^2^/g and a total pore volume of 0.039 cm^3^/g, indicating limited volatile matter release and minimal pore development [28]. In contrast, increasing the temperature to 700 °C (CB700) resulted in substantial enhancement, increasing the surface area to 333.0 m^2^/g and pore volume to 0.187 cm^3^/g. This improvement was attributed to the intensified thermal decomposition of the organic matrices at higher temperatures, which promoted the collapse of the dense structures and the formation of a porous carbon skeleton. These structural transformations are consistent with previous findings that increased pyrolysis temperatures facilitate the development of hierarchical pore systems in biochar [29].

Water washing further enhanced the textural characteristics of the biochar. For WCB500 and WCB700, the BET surface area increased to 69.5 and 378.3 m^2^/g, respectively, with slight increases in pore volume. These improvements likely resulted from the removal of ash and mineral residues that impeded access to the internal pores, as supported by studies indicating that post-treatment enhances pore accessibility. The slight reduction in the average pore diameter after washing indicates increased exposure to smaller mesopores and micropores, which is associated with the removal of pore-blocking contaminants [30].

Overall, the synergistic effect of high-temperature pyrolysis and water washing yielded biochars with a high surface area and well-developed porosity, which are critical for enhancing the adsorption performance for organic pollutants such as Rhodamine B. These findings are consistent with the broader biochar modification studies demonstrating that thermal and chemical treatments can adjust pore structures for specific environmental applications.

#### 2.1.3. XRD Analysis

**Figure 2:**
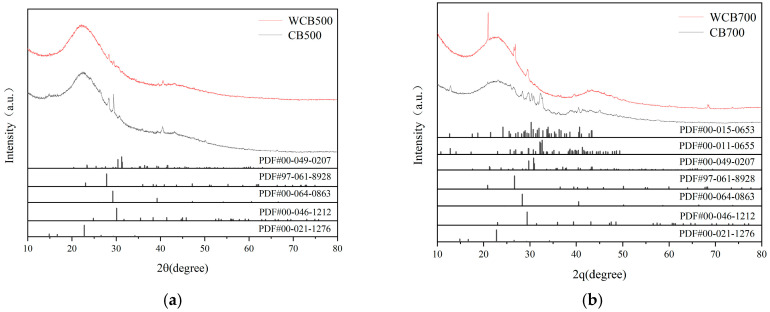
X-ray diffraction shows reduced mineral peaks after washing and high-temperature pyrolysis. (**a**) XRD Comparison; (**b**) XRD Patterns of WCB700 and CB700.

XRD was employed to investigate the crystallographic composition and mineralogical evolution of the biochar samples by leveraging its nondestructive capability to characterize crystal structures and quantify mineral phases [31]. This technique, which is extensively used in materials science and mineralogy, facilitates the accurate identification of crystalline phases by comparing them with standard diffraction patterns such as those found in the JCPDS database (Figure 2). It provides critical insights into the thermal and chemical transformations during pyrolysis and post-treatment [32].

For the 500 –pyrolyzed samples (CB500 and WCB500), distinct diffraction peaks at 2θ = 28.4°, 40.2°, and 50.1° were attributed to potassium chloride (KCl) and calcium carbonate (CaCO_3_), with weaker signals from potassium sulfate (K_2_SO_4_) and silica (SiO_2_). These minerals originate from the thermal decomposition of alkali and alkaline earth metals in coffee shell biomass, which is consistent with findings for similar feedstocks [33]. Water washing (WCB500) significantly reduced peak intensities, particularly for SiO_2_, indicating effective removal of water-soluble salts and amorphous silica through dissolution. This is consistent with studies indicating that aqueous post-treatment can remove up to 80% of the loosely bound mineral residues, thereby enhancing biochar purity [32].

At 700 °C, CB700 and WCB700 exhibited broadened diffraction patterns, indicating reduced crystallinity and the formation of thermally stable minerals such as K_2_CO_3_·1.5H_2_O. Although CB700 retained residual peaks for KCl, K_2_SO_4_, and CaCO_3_, their intensities were lower than those in 500 °C samples owing to partial decomposition and structural rearrangement at high temperatures [33]. Water washing of CB700 (WCB700) further minimized mineral signals, resulting in only weak reflections for CaCO_3_ and K_2_SO_4_. This underscores the synergistic effect of increased pyrolysis temperature and aqueous treatment in reducing mineral impurities.

The broad hump at 2θ = 20–30° in all samples, which indicates the presence of amorphous carbon and graphitized structures, became pronounced in WCB700, suggesting enhanced structural ordering. This observation is consistent with the understanding that high-temperature pyrolysis promotes the condensation of aromatic domains, a process facilitated by the removal of mineral obstructions during washing [31,33]. The combined results demonstrate that XRD effectively monitors mineralogical changes induced by the pyrolysis temperature and post-treatment, providing a foundation for optimizing biochar synthesis for environmental applications.

The condensation of aromatic domains during high-temperature pyrolysis is beneficial for enhancing the structural stability and aromaticity of the biochar, which increases its adsorption capacity. However, this process requires significant energy input, which could affect the overall cost-effectiveness of high-temperature pyrolysis. Therefore, the benefits of improved adsorption should be weighed against the energy required for such high-temperature treatments.

#### 2.1.4. FTIR Analysis

**Figure 3:**
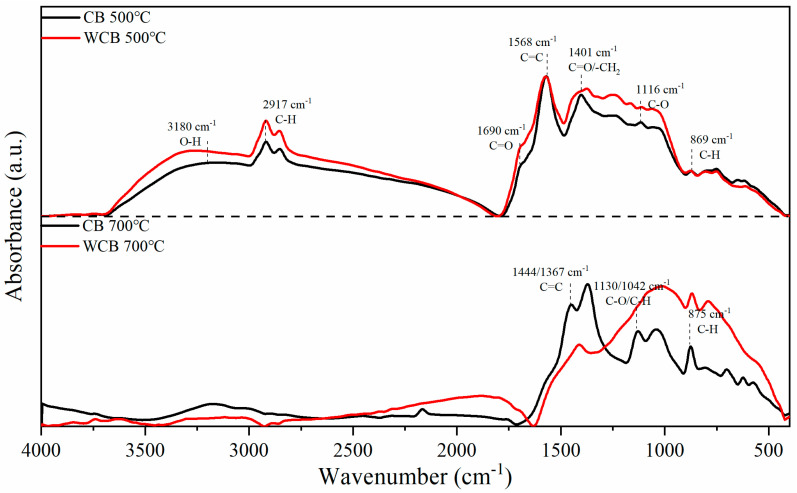
Infrared spectrum characteristics of coffee shell biochar samples prepared at different temperatures before and after washing.

Figure 3 shows the infrared spectral features of the coffee shell-derived biochar samples prepared at different pyrolysis temperatures and washed with water. A broad absorption band observed near 3180 cm^−1^ in the samples carbonized at 500 °C corresponds to the O–H stretching vibration, which is characteristic of hydroxyl-containing species. Absorption above 3500 cm^−1^ is attributed to free O–H groups, while the region below 3500 cm^−1^ is mainly associated with hydrogen-bonded hydroxyls from alcohols and phenols. The bands in the 3000–2800 cm^−1^ region are attributed to the C–H stretching vibrations of methyl and methylene groups, primarily originating from residual aliphatic hydrocarbons and cycloalkane structures in the biochar matrix.

The prominent peak at 1568 cm^−1^ is within the characteristic aromatic region and is associated with the C=C stretching of benzene rings. A shoulder peak at approximately 1690 cm^−1^ is commonly attributed to the stretching vibration of carbonyl groups (C=O). The band at 1401 cm^−1^ is relatively complex and may involve CH_3_/CH_2_ bending, symmetric C=O stretching, and other related modes. The peak at approximately 1116 cm^−1^ results from C–O stretching in phenols and alcohols, as well as C–O–C vibrations in carbohydrates. Additional contributions may include CH_3_ rocking, C–C stretching in aliphatic chains, and in-plane bending of aromatic C–H bonds. The absorption at approximately 869 cm^−1^ is attributed to the out-of-plane bending vibration of aromatic C–H groups on substituted side chains.

With increasing pyrolysis temperature to 700 °C, the O–H stretching band at approximately 3180 cm^−1^ and the aliphatic C–H stretching bands in the 3000–2800 cm^−1^ region completely disappeared. This indicated the occurrence of extensive thermal decomposition, decarboxylation, and dehydration reactions, leading to the formation of more condensed aromatic domains and graphitized carbon structures. Notably, the peaks at 1444 and 1367 cm^−1^, corresponding to aromatic C=C stretching, were more pronounced after high-temperature pyrolysis. Simultaneously, the relative intensities of the C–O stretching bands at 1130 and 1024 cm^−1^ were notably reduced, indicating the removal of oxygen-containing surface functionalities and an increase in aromatic character.

Washing with water induced further changes in the FTIR spectra. The removal of soluble inorganic salts and mineral residues effectively reduced interference during pyrolysis, thereby facilitating more conversion of organic matter into aromatic carbon structures. In addition, an increase in the relative organic matter content during pyrolysis enhanced the degree of aromatization of the resulting biochar. Specifically, for WCB700, a broad absorption feature at approximately 1000 cm^−1^ was observed, which potentially indicates the formation of a more ordered graphite-like lattice after the removal of inorganic components. Furthermore, a slight increase in peak intensity was detected in the 700–900 cm^−1^ region, corresponding to out-of-plane C–H bending of aromatic structures, likely owing to reduced byproduct accumulation and enhanced retention of aromatic carbon frameworks and side-chain functionalities.

In summary, washing with water effectively promotes the carbonization process by removing inorganic impurities, resulting in a considerably stable and aromatized carbon matrix. For biochar prepared at 500 °C, the spectral differences before and after washing were less pronounced, likely because of the limited degree of carbonization at low temperatures; the material retained substantial amounts of cellulose, hemicellulose, and lignin, as well as abundant oxygen-containing functional groups.

The development of hierarchical pore structures observed in the SEM images, particularly in WCB700, correlates with the large increase in surface area and pore volume measured by BET. This relationship suggests that the pyrolysis temperature and post-treatment washing enhance the biochar’s porosity, providing more active sites for adsorption.

#### 2.1.5. Chemical Composition: Ash, Volatile Matter, and Moisture Content

In Table 2, the ash content, volatile matter content, and moisture content of coffee shell biochar samples are presented. These properties were obtained through analysis of biochars prepared at two different pyrolysis temperatures (500 °C and 700 °C) (CB500, WCB500, CB700, and WCB700). The data show a significant reduction in ash content after washing, particularly in high-temperature-treated biochars (CB700 to WCB700), indicating that washing effectively removes residual ash. The reduction in ash content is crucial as it may expose more active sites on the biochar surface, potentially improving its adsorption performance, as discussed further in this paper.

### 2.2. Adsorption Kinetics: Rate Analysis and Model Fitting

#### Adsorption Kinetics

**Figure 4:**
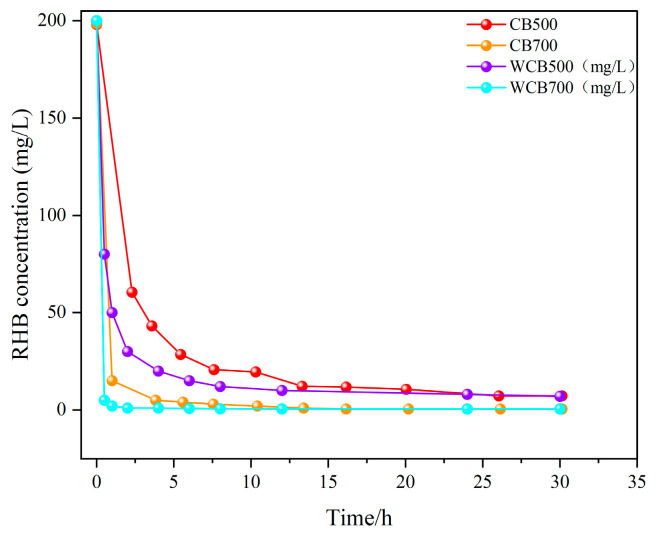
Kinetic curves of Rhodamine B (RhB) removal by biochar at different pyrolysis temperatures.

Understanding the adsorption kinetics is essential for evaluating the rate and mechanism of RhB removal by biochar. In this study, kinetic experiments were conducted using biochars produced at different pyrolysis temperatures (500 and 700 °C), with and without water-washing treatment. The temporal concentration profiles of the RhB in solution are shown in Figure 4.

Figure 4 shows that all biochar samples exhibited a two-stage adsorption behavior: an initial rapid adsorption phase occurring within the first 1–2 h, followed by a slower progression towards equilibrium. This pattern is characteristic of many porous adsorbents and indicates that the availability of abundant active sites and rapid surface interactions controls the initial phase. The adsorption rate decreased over time owing to the occupation of the available sites and increased mass transfer resistance.

Notably, the high-temperature samples (CB700 and WCB700) demonstrated significantly enhanced adsorption kinetics compared with those of the low-temperature samples (CB500 and WCB500). WCB700 removed almost all RhB within 1 h, whereas CB500 showed a slower uptake and lower removal efficiency during the test duration. These results indicate that higher pyrolysis temperature and post-washing treatment enhance adsorption performance by increasing specific surface area, pore volume, and exposure of functional groups.

To further elucidate the adsorption mechanisms, the experimental kinetic data were fitted using pseudo-first-order and pseudo-second-order models, as described by the following equation:

Ln(q_e_ − q_t_) = ln (q_e_) − k_1_ t (1)

where q_t_ (mg/g) is the amount of RhB adsorbed at time t (h), q_e_ (mg/g) is the adsorption capacity at equilibrium, and k_1_ (h^−1^) and k_2_ (g·mg^−1^·h^−1^) are the rate constants of the respective models. The kinetic parameters obtained from the fitting are presented in Table 3.

The pseudo-second-order model consistently exhibited higher correlation coefficients (R^2^ > 0.98) than those of the pseudo-first-order model, indicating that chemisorption was the dominant adsorption mechanism. Chemisorption involves stronger interactions, such as electron sharing or exchange between RhB molecules and the active sites on the biochar surface. This includes π–π stacking between aromatic domains and the dye, as well as hydrogen bonding via oxygen-containing groups.

Among all the samples tested, WCB700 exhibited the highest adsorption capacity (qe = 147.6 mg/g) and the fastest kinetic rate constant (k_2_ = 0.0171 g·mg^−1^·h^−1^), indicating its superior porosity and surface reactivity. The substantial improvements after washing were attributed to the removal of surface ash and inorganic blockages, which exposed the active adsorption sites and facilitated solute diffusion into the internal pores.

These results indicate that the combination of pyrolysis temperature and water washing considerably improves the kinetic performance of biochar in the absorption of organic dye. The predominance of pseudo-second-order kinetics further indicates that surface chemical interactions primarily influence the adsorption process rather than simple physical diffusion.

In the experiment, changes in ash content significantly affected the adsorption performance of biochar. Especially in high-temperature pyrolysis samples, the ash content after water washing (WCB700) was notably reduced, thereby increasing the active surface sites of the biochar. The ash content of the CB700 sample was 14.8%, while that of the water-washed WCB700 sample dropped to 7.2%. This change significantly enhanced its adsorption capacity (from 193.5 mg/g to a higher value), indicating that removing ash helps improve the adsorption performance of biochar. This may be because the removal of ash components exposes more active sites, thus enhancing the adsorption effect.

However, this study primarily focuses on the adsorption performance of Rhodamine B (RhB). However, the combination of pyrolysis temperature and water washing significantly improved the kinetic efficiency of biochar, an effect that is expected to not be limited to RhB. We believe that similar pyrolysis and water-washing treatments may also have a similar promoting effect on other organic pollutants. High-temperature pyrolysis and water washing improved the pore structure and active surface sites of biochar, which may enhance its adsorption capacity for other pollutants. Additionally, differences in the molecular structure and hydrophilicity or hydrophobicity of other organic pollutants may also affect the adsorption process. Therefore, to verify whether these effects apply to other substances, future research should include evaluations of the adsorption performance of different pollutants, such as common dyes, pesticides, and heavy metal ions. Through these further studies, the scope of application for optimizing biochar through pyrolysis temperature and water washing can be expanded, making it an effective technology widely applicable to the removal of various environmental pollutants.

### 2.3. Effects of Pyrolysis Temperature, Washing Treatment, and Solid-to-Liquid Ratio on Adsorption

**Figure 5:**
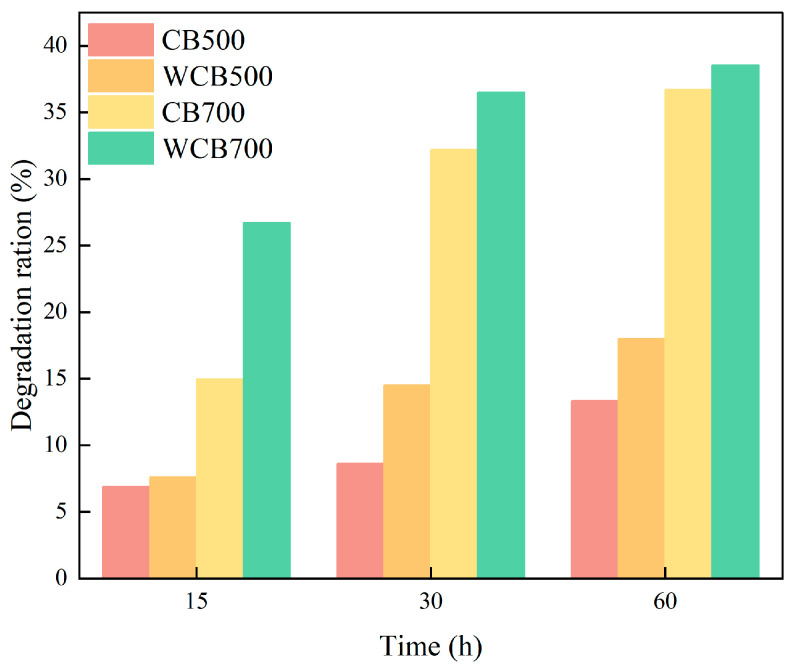
Degradation rates of Rhodamine B (RhB) by biochar with different carbonization temperatures before and after washing at different reaction times.

The pyrolysis temperature significantly affected biochar adsorption performance. CB700 exhibited a higher adsorption capacity for RhB than that of CB500, mainly because of its considerably developed pore structure and larger specific surface area. Washing increased biochar adsorption capacity. WCB500 and WCB700 exhibited higher adsorption capacities than those of CB500 and CB700, respectively, because the washing treatment removed some impurities from the biochar surface and exposed more active sites (Figure 5).

The solid–liquid ratio typically affects the apparent removal rate of RhB by biochar. Biochar exhibited a similar trend, with the removal rate of biochar RhB increasing as the solid-to-liquid ratio increased.

**Figure 6:**
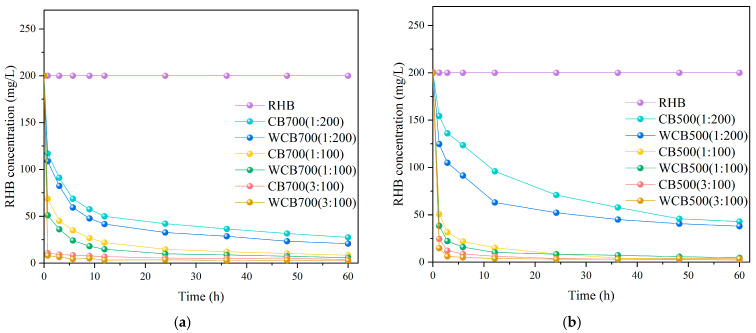
Effect of solid-to-liquid ratio on the degradation rate of Rhodamine B (RhB) by biochar. (**a**) RhB Removal by 700 °C Samples (CB700/WCB700); (**b**) RhB Removal by 500 °C Samples (CB500/WCB500).

The degradation rates of biochar and washed biochar RhB with different solid–liquid ratios were quantified separately. For the 500 °C sample, biochar and washed biochar had the highest solid–liquid ratio at 1:100 (Figure 6a). For the 700 °C sample, an increase in biochar content resulted in a higher degradation rate of RhB (Figure 6b). The solid-to-liquid ratio also affected the adsorption process. As the solid-to-liquid ratio increased, the adsorption capacity of the biochar initially increased and then stabilized. This is because a higher solid-to-liquid ratio provides more adsorption sites. However, when the solid-to-liquid ratio is too high, agglomeration of biochar particles occurs, reducing the effective adsorption surface area.

From a molecular diffusion perspective, increasing the solid–liquid ratio changes the relative concentration of RhB in the system. When the solid–liquid solution was relatively low, the solution had relatively more RhB molecules and higher RhB concentrations around the biochar. As the solid–liquid ratio and biochar content increased, the RhB molecules in the solution required diffusion to additional biochar surfaces for adsorption. The diffusion principle states that the concentration gradient is the driving force of diffusion. An increase in the solid–liquid ratio increased the gradient of the RhB concentration between the surface of the biochar and the main body of the solution. This large concentration gradient accelerated the diffusion rate of RhB molecules to the surface of the biochar and then the adsorption process, which increased the removal rate of RhB by the biochar.

The adsorption of RhB by biochar gradually reached equilibrium. At higher solid–liquid ratios, more RhB molecules can be adsorbed rapidly owing to the increased availability of adsorption sites and larger concentration gradients, thereby facilitating the faster establishment of the adsorption equilibrium. Figure 6 shows that the curve under the high solid–liquid ratio condition tends to flatten in a short time, indicating that it reaches adsorption equilibrium faster. This demonstrates the characteristics of faster removal speed.

### 2.4. Regeneration and Reusability

The regeneration ability of an adsorbent is a critical factor for assessing its potential for practical and sustainable wastewater treatment applications. In this study, the reusability of the biochars was evaluated through consecutive adsorption–desorption cycles using RhB as the model pollutant. WCB700, which exhibited the highest initial adsorption performance, was selected as a representative sample for the regeneration experiment.

After each adsorption cycle, the spent biochar was recovered by filtration and subsequently desorbed using a 0.1 mol/L NaOH solution or ethanol with mild stirring. The adsorbent was then thoroughly washed with deionized water, dried at 80 °C, and reused under identical conditions for up to five cycles. The residual RhB concentration was recorded for each cycle, and the regeneration efficiency was calculated based on the initial adsorption capacity.

**Figure 7:**
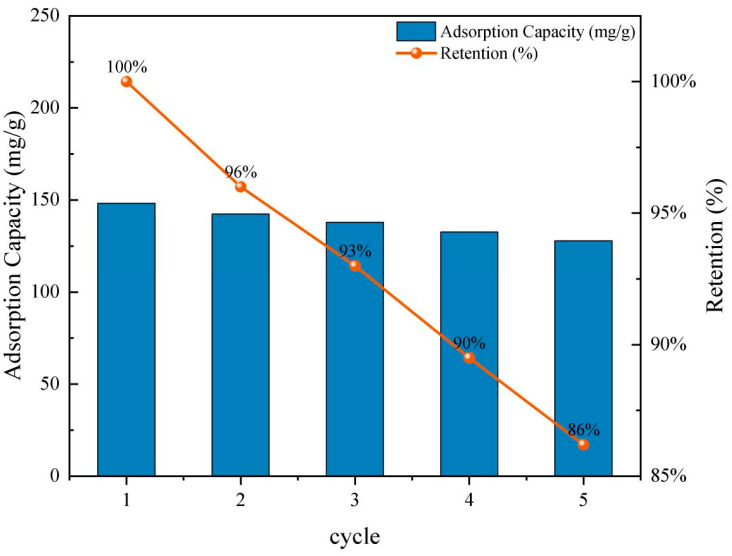
Recycling experiment.

Figure 7 shows that WCB700 maintained a high removal efficiency across all five cycles. The adsorption capacity slightly decreased from 148.2 mg/g in the first cycle to 127.8 mg/g in the fifth cycle, resulting in a retention rate of approximately 86.2%. This moderate decline is attributed to partial pore blockage or irreversible binding of RhB molecules to high-affinity sites that are not easily desorbed under mild conditions.

The excellent regeneration performance was closely related to the robust carbon structure formed at high pyrolysis temperatures, as well as the removal of inorganic residues by water washing, which maintained accessible adsorption sites and reduced surface fouling. Additionally, the presence of abundant aromatic carbon domains and oxygen-containing functional groups may facilitate reversible π–π interactions and hydrogen bonding with dye molecules, thereby enabling efficient desorption.

The WCB700 biochar demonstrated outstanding reusability and structural stability, confirming its potential as a cost-effective and recyclable adsorbent for organic dye removal in repeated batch operations.

After five regeneration cycles, the WCB700 biochar retains more than 85% of its initial adsorption capacity, indicating its excellent reusability. This demonstrates that WCB700 can be safely regenerated multiple times without significant loss of efficiency, making it a viable option for long-term use in water treatment applications.

### 2.5. Adsorption Mechanism

A comprehensive understanding of the adsorption mechanism is essential to elucidate the interaction pathways between RhB molecules and the biochar surface. Based on the results from material characterization (SEM, BET, XRD, and FTIR), including kinetic and isotherm analyses, the adsorption of RhB onto the prepared biochars is proposed to be influenced by a combination of physical and chemical interactions, such as π–π electron donor–acceptor interactions, hydrogen bonding, electrostatic attraction, and pore filling.

#### 2.5.1. Surface Morphology and Porosity

The SEM images revealed a substantial improvement in pore development after pyrolysis, particularly at 700 °C. Water washing further removed residual ash and block particles, resulting in cleaner and more accessible pore structures [34]. BET analysis (confirmed a substantial increase in specific surface area and pore volume for WCB700, which facilitated greater surface accessibility and faster mass transfer. These structural advantages are consistent with the rapid adsorption observed in the kinetic studies, particularly for the high-temperature water-washed samples.

The large surface area and mesoporous structure primarily contributed to the pore-filling effect, facilitating RhB molecules to diffuse into the biochar matrix. This mechanism was prominent at higher dye concentrations, as indicated by the good fit of the Langmuir model.

#### 2.5.2. Surface Functional Groups

The FTIR spectra indicated the presence of hydroxyl (–OH), carbonyl (C=O), and aromatic (C=C) groups in all samples, with a considerable reduction in oxygenated functionalities at higher pyrolysis temperatures [35]. In particular, the C=C aromatic ring signals were more prominent after carbonization at 700 °C, and water washing further exposed these graphitized domains.

These structural features indicate that π–π interactions played a dominant role in the adsorption process, where the conjugated π-system of the aromatic domains in biochar interacts with the aromatic ring of RhB. This is further supported by the increased adsorption capacity and accelerated kinetics observed for WCB700, which exhibited a higher graphitic content [36].

Meanwhile, residual oxygen-containing groups such as –OH and –COOH, although reduced at high temperatures, can still participate in hydrogen bonding with the polar groups of RhB, such as amino and carbonyl groups, increasing the affinity at specific sites [37].

#### 2.5.3. Electrostatic Interactions

At the pH used in the adsorption experiments (typically near neutral), RhB predominantly existed as a cationic dye. Although the surface charge of biochar is generally negative owing to deprotonated functional groups, this charge can vary with temperature and surface treatment. Washing with water removes surface metal ions and soluble salts, thereby increasing the net negative surface potential. This promotes electrostatic attraction between the positively charged RhB molecules and the negatively charged biochar surface. This mechanism is crucial in WCB500 and WCB700, where inorganic impurities are primarily removed, notably exposing the functional sites.

#### 2.5.4. XRD Insights: Role of Residual Minerals

XRD patterns showed that unwashed biochars contained crystalline minerals such as KCl, CaCO_3_, and SiO_2_. These components may block pores, alter the surface charge, or interfere with dye–adsorbent interactions. Washing with water effectively removes these residues, improving structural homogeneity and eliminating potential competition at the adsorption interface. Therefore, mineral removal indirectly enhances π–π interaction, electrostatic attraction, and pore accessibility by revealing the true adsorption-active surfaces [38].

These findings demonstrate that the adsorption of RhB onto the prepared coffee shell-derived biochars is a synergistic process driven by well-developed pore structures, graphitized surface domains, and active functional groups exposed through optimized pyrolysis and water washing. The material developed in this study exhibited competitive or superior performance regarding adsorption capacity, regeneration efficiency, and mechanistic diversity compared with other reported biochar-based adsorbents.

To contextualize these results, Table 4 presents a comparative summary of this study and several representative studies from the literature. This comparison outlines the differences in raw material selection, structural engineering strategies, maximum adsorption capacities, and mechanistic insights, highlighting the unique advantages of the current biochar system.

## 3. Materials and Methods

### 3.1. Material Preparation

Coffee shell samples used in this study were collected from local coffee shops. The composition of the original coffee shell biomass includes 35.3% cellulose, 22% lignin, and 10.5% ash, which are consistent with the typical composition of coffee waste in the literature, indicating that it has high availability as a raw material for biochar. After collection, the samples were immediately washed with deionized water to remove dust and surface impurities, which is a standard procedure used to ensure the removal of contaminants [41]. The cleaned coffee shells were dried in an oven at 105 °C for 12 h to reduce the moisture content to ˂5%, thereby optimizing them for pyrolysis [42]. Subsequently, the dried coffee shells were ground into a powder using a grinder and sieved through a 60-mesh sieve to obtain a uniform particle size, facilitating consistent pyrolysis and adsorption characteristics [43].

### 3.2. Biochar Preparation Process

Biochar was prepared by slow anaerobic pyrolysis in a tube furnace, a process that enhances carbon retention and the formation of porous structures [44]. A specific amount of coffee shell powder was placed in a crucible in a tube furnace. Nitrogen gas was continuously supplied to create an oxygen-free environment, prevent combustion, and promote pyrolysis [45]. After purging for 30 min, the furnace was heated at a rate of 15 °C/min to the target temperatures of 500 and 700 °C [46]. The samples were maintained at these temperatures for 2 h to ensure complete pyrolysis and cooled to room temperature within the furnace under a nitrogen atmosphere to prevent oxidation [47]. The resulting biochar samples were labelled CB500 and CB700, corresponding to their respective pyrolysis temperatures.

To evaluate the effect of washing on biochar performance, the CB500 and CB700 samples were washed with ultrapure water until the pH of the washing solution stabilized. This process removes residual inorganic compounds and enhances adsorption capacity [48]. The washed biochar samples were then dried at 60 °C for 24 h to remove the residual moisture without altering their surface characteristics [49]. These samples were designated WCB500 and WCB700, respectively.

### 3.3. Material Characterization Methods

The surface morphologies of the biochar samples were examined using SEM (JEOL-JSM7401F, JEOL Ltd., London, UK), a technique widely employed to observe the porous structures [50]. Prior to imaging, the samples were coated with a thin layer of gold to enhance conductivity and image resolution [51]. The surface morphology was analyzed using scanning electron microscopy. The standard deviation in pore size measurements is ±5%.

The specific surface area and pore structure were analyzed by a porosity and surface area analyzer (ASAP 2020, Micromeritics Instrument Corporation, Norcross, GA, USA) using the BET method [52]. The specific surface area was measured using the Brunauer–Emmett–Teller (BET) method. The error in surface area measurement is ±3%.”

FTIR (JASCO FT-IR-4700, JASCO Corporation, Tokyo, Japan) was used to identify the functional groups on the biochar surface, providing information on the chemical functionalities that interact with the adsorbates [53]. Spectra were recorded over a wavenumber range of 400–4000 cm^−1^ to capture a comprehensive profile of surface functionalities [54]. Fourier transform infrared spectroscopy was used to analyze the surface functional groups. The spectral resolution is ±1 cm^−1^.

X-ray photoelectron spectroscopy (ULVAC-PHI, PHI 5000 VersaProbe, ULVAC-PHI, Inc., Chigasaki, Japan) was employed to analyze the elemental composition and chemical states of the biochar before and after adsorption. This analysis provides detailed information on the surface chemistry and potential interaction mechanisms [55].

The specific surface area and pore structure were analyzed by a porosity and surface area analyzer (ASAP 2020, Micromeritics Instrument Corporation, Norcross, GA, USA) using the BET method. The surface area measurements were conducted in accordance with IUPAC recommendations for gas adsorption analysis based on the Brunauer–Emmett–Teller (BET) theory. Pyrolysis of the coffee shell biomass was carried out under limited oxygen conditions, following the ASTM D1762-84 standard for the chemical analysis of wood charcoal, to ensure reproducibility and comparability with other biochar studies [56].

### 3.4. Adsorption Experiment

A stock solution of RhB with an initial concentration of 200 mg/L was prepared by dissolving an appropriate amount of RhB powder in deionized water to ensure consistency under the experimental conditions [57]. Adsorption experiments were conducted in a series of conical flasks, each containing a specific amount of biochar added to RhB solutions of varying initial concentrations (5–200 mg/L) at a constant temperature of 25 ± 0.5 °C [58].

All adsorption experiments, including kinetic tests, were conducted in triplicate under identical conditions. The data presented in the kinetic plots represent the average values of three independent experiments. Although error bars are not shown in the figures, the relative standard deviation for most data points was found to be below 5%, indicating high reproducibility.

The mixtures were agitated on an orbital shaker at 150 rpm to maintain a uniform suspension and facilitate contact between the adsorbent and adsorbate [59]. At predetermined intervals, aliquots of the supernatant were extracted and filtered through a 0.45-μm membrane to remove biochar particles [60]. The RhB concentration in the filtrate was determined using a UV-visible spectrophotometer at a wavelength of 554 nm, which corresponds to the maximum absorbance of RhB, to quantify the adsorption capacity [61]. The adsorption capacity (mg/g) of RhB on biochar at a particular time t was calculated using the following formula:

(2) $$q_{t}=\frac{(C_{0}-C_{t})V}{m}$$

where *C*_0_ and *C*_t_ are the initial and time-t concentrations of RhB in the solution (mg/L), respectively; *V* is the volume of the solution (L); and m is the mass of the biochar (g).

The removal efficiency (*R*, %) of RhB was calculated as follows:

(3) $$R=\frac{C_{0}-C_{t}}{C_{0}}\times 100\%$$

To study the effect of pH on the adsorption process, the initial pH of the RhB solution was adjusted to different values (2–10) using 0.1 M HCl or 0.1 M NaOH before adding the biochar. The influence of the solid-to-liquid ratio on adsorption was investigated by varying the amount of biochar added to a fixed volume of RhB solution.

### 3.5. Adsorption Kinetics and Isotherm Models

The adsorption kinetics data were fitted to pseudo-first-order and pseudo-second-order models. The pseudo-first-order model is expressed as follows:

(4) $$q_{t}=q_{e}(1-e^{-k_{1}t})$$

where *q_e_* is the equilibrium adsorption capacity (mg/g), *k*_1_ is the rate constant of the pseudo-first-order model (min^−1^), and *t* is the adsorption time (min).

The pseudo-second-order model is expressed as follows:

(5) $$q_{t}=\frac{k_{2}{q^{2}}_{e}t}{1+k_{2}qet}$$

where *k*_2_ is the rate constant of the pseudo-second-order model (g/mg·min).

The adsorption isotherm data were fitted using the Langmuir and Freundlich models. The Langmuir model is expressed as follows:

(6) $$\frac{1}{q_{e}}=\frac{1}{q_{m}}+\frac{1}{k_{L}q_{m}C_{e}}$$

where *q_m_* is the maximum adsorption capacity (mg/g), *k_L_* is the Langmuir adsorption constant (L/mg), and *C_e_* is the equilibrium concentration of RhB in the solution (mg/L).

The Freundlich model is expressed as follows:

(7) $$Inq_{e}=InK_{F}+\frac{1}{n}InC_{e}$$

where *K_F_* is the Freundlich adsorption constant related to the adsorption capacity, and *n* is an empirical parameter reflecting the adsorption intensity.

### 3.6. Regeneration Study

**Scheme 1:**
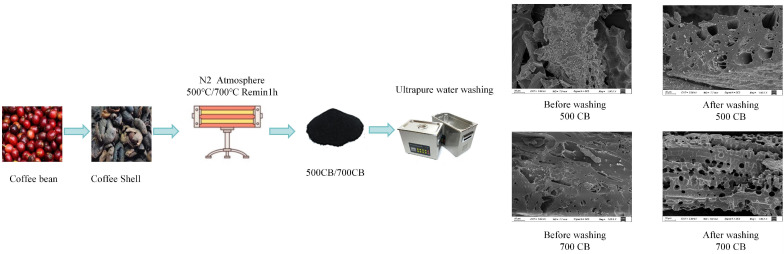
Preparation of coffee shell biochar.

Regeneration experiments were conducted after the adsorption of RhB to demonstrate the reusability of biochar. After each adsorption cycle, the RhB-loaded biochar was regenerated by washing with a desorbing agent, specifically 0.1 M HCl or 0.1 M NaOH [62]. The regenerated biochar was dried at 60 °C and reused in subsequent adsorption cycles. The adsorption capacity and removal efficiency of the regenerated biochar were measured at each cycle to evaluate its reusability (Scheme 1).

## 4. Conclusions

This study comprehensively investigated the synthesis, structural changes, and adsorption performance of biochars derived from coffee shells, a low-cost and abundant agricultural waste product. Through a controlled pyrolysis process and subsequent water-washing treatment, we demonstrated that the surface chemistry, porosity, and functional accessibility of biochar can be finely modulated to achieve highly efficient adsorption of RhB.

All experimental data have taken into account the measurement error and confidence interval. The error of surface area and porosity measurement is ±3% and ±5%, and the confidence interval of adsorption capacity is ±3%. The reliability of these data provides strong support for the conclusions of this paper.

These findings indicate that structural optimization involves increasing the surface area as well as a detailed balance between aromatic condensation, removal of mineral residues, and activation of adsorption sites. The superior performance of WCB700 resulted from this synergistic approach, indicating a transition from disordered carbon to semigraphitized surfaces that are highly accessible for π–π interactions, hydrogen bonding, and electrostatic adsorption.

Kinetic and isothermal analyses further supported this interpretation, with pseudo-second-order and Langmuir models revealing chemisorption-driven monolayer adsorption. Notably, WCB700 retained more than 85% of its adsorption capacity after five cycles, highlighting its structural resilience and practical viability.

However, this study has limitations. The experimental conditions employed, such as single-solute systems, constant pH, and batch adsorption, do not entirely represent the complexities encountered in real-world scenarios. Future studies should include multi-contaminant systems, dynamic flow environments, and long-term stability tests to evaluate performance robustness. Additionally, theoretical models such as the density functional theory simulations can enhance our understanding of interfacial interactions.

This study demonstrates that both pyrolysis temperature and water-washing treatment significantly affect the properties and adsorption performance of coffee shell-derived biochar. Notably, water washing effectively removes ash content from the biochar, exposing more active sites on the surface. This reduction in ash content is crucial for enhancing the adsorption capacity of the biochar, making it more efficient for removing contaminants such as Rhodamine B from aqueous solutions. These findings provide valuable insights into optimizing biochar for environmental applications, especially in wastewater treatment.

## Figures and Tables

**Table 1:** Brunauer–Emmett–Teller-specific surface area, total pore volume, and average pore size of biochar samples prepared under different conditions.

Sample	Temperature (℃)	Washing	BET Surface Area (m^3^/g)	Pore Volume (cm^3^/g)	Average Pore Size (nm)
CB500	500	No	56.3	0.039	2.77
WCB500	500	Yes	69.5	0.046	2.65
CB700	700	No	333	0.187	2.25
WCB700	700	Yes	378.3	0.205	2.16

**Table 2:** Ash and moisture content of the biochar.

Sample	Temperature (°C)	Ash Content (%)	Volatile Matter (%)	Moisture Content
Coffee Shell	-	10.5	70.1	5.0
CB500	500	12.3	64.2	4.7
CB700	500	9.1	67.5	3.9
WCB500	700	14.8	60.1	4.4
WCB700	700	7.2	62.3	3.5

**Table 3:** Kinetic model fitting parameters for Rhodamine B (RhB) adsorption.

Sample	Model	qe (mg/g)	k (Rate Constant)	R^2^
CB500	Pseudo-first-order	85.4	0.092 h^−1^	0.882
CB500	Pseudo-second-order	93.7	0.0047 g·mg^−1^·h^−1^	0.983
CB700	Pseudo-first-order	121.1	0.121 h^−1^	0.897
CB700	Pseudo-second-order	133.2	0.0118 g·mg^−1^·h^−1^	0.996
WCB500	Pseudo-first-order	103.3	0.101 h^−1^	0.913
WCB500	Pseudo-second-order	115.9	0.0075 g·mg^−1^·h^−1^	0.99
WCB700	Pseudo-first-order	138.8	0.143 h^−1^	0.921
WCB700	Pseudo-second-order	147.6	0.0171 g·mg^−1^·h^−1^	0.998

**Table 4:** Comparison of the present biochar with previously reported biochars for Rhodamine B adsorption.

Contrast Dimensions	This Study	[39]	[40]
Raw materials and preparation methods	Low-cost recycling of agricultural waste (coffee shell); only pyrolysis (500/700 °C) + water washing, no chemical activator, and an environmentally friendly process.	Coconut shell requires Fe/N co-doping (urea+FeSO_4_), complex chemical modification, and potential secondary contamination risk.	Waste cotton needs KOH high-temperature activation (800 °C), high energy consumption, large KOH consumption (mass ratio 1:5), and high cost.
Adsorption capacity; loading capacity	193.5 mg/g (RhB) to meet the actual wastewater treatment needs and balance the adsorption performance and cost.	12.41 mg/g (RhB), with low capacity, requiring multiple adsorptions or increased dosage.	Ultra-high capacity (7265 mg/g CR), but requires very high pollutant concentration (700 mg/L), limited practical application.
Adsorption mechanism	Comprehensive mechanism analysis (π-π stacking, hydrogen bonding, electrostatic, pore filling), through multiple characterization verification, is highly scientific.	Dependent on Fe/N doping to enhance chemical adsorption, but the mechanism validation, that is, the Fe-N bond role, has not been thoroughly explored.	Depends on physical adsorption (pore filling + van der Waals force) with weak chemical action and limited applicability to complex systems.
Regenerative performance	After five cycles, 85% of the capacity is retained, only NaOH/ethanol desorption is required, the cost of regeneration is low, and stability is excellent.	After five cycles, the capacity is reduced to 43.8%, combining the H_2_O_2_ oxidation recovery performance (complex operation and may destroy the structure).	Mass loss of 87% (50 mg; 6.63 mg) after three cycles, calcination + KOH for regeneration, high energy consumption, and not sustainable.
Theoretical contribution	To reveal the synergistic effect of pyrolysis temperature and washing and to provide a universality strategy for chemical optimization of biochar surface.	The Fe-N co-modification improves the adsorption capacity, but it is limited to a single RhB pollutant and lacks universality.	Activated carbon with a high specific surface area was developed, but the problem of regeneration was not solved, and its practical application value is limited.
Environmental protection and economy	Entirely based on waste + no chemical activation, in accordance with the circular economy; simple process, easy to large-scale production.	Relying on chemical modification (FeSO_4_/urea), Fe/N residue may be introduced in wastewater treatment.	KOH activation requires a large amount of strong alkali, high cost + high environmental burden, and low feasibility of industrialization.
Application potential	Suitable for medium and low concentrations of dye wastewater (with the initial RhB concentration of 200 mg/L), with stable regeneration performance, and suitable for long-term recycling use.	Magnetic separation is suitable for heavy metal or complex ion systems but has a low adsorption capacity and narrow application scenarios.	Only applicable to ultra-high concentration wastewater (such as industrial dye waste liquid), high regeneration cost is difficult to popularize.

## Data Availability

The data presented in this study are available in the article and can be shared upon request.

## Abbreviations

BET	Brunauer–Emmett–Teller method
CSB	Coffee shell-derived biochar
FTIR	Fourier transform infrared spectroscopy
RhB	Rhodamine B
SEM	Scanning electron microscopy
XRD	X-ray diffraction
